# Hyaluronic Acid in the Third Millennium

**DOI:** 10.3390/polym10070701

**Published:** 2018-06-25

**Authors:** Arianna Fallacara, Erika Baldini, Stefano Manfredini, Silvia Vertuani

**Affiliations:** Department of Life Sciences and Biotechnology, Master Course in Cosmetic Science and Technology (COSMAST), University of Ferrara, Via L. Borsari 46, 44121 Ferrara, Italy; arianna.fallacara@student.unife.it (A.F.); erika.baldini@student.unife.it (E.B.); vrs@unife.it (S.V.)

**Keywords:** biological activity, crosslinking, drug delivery, cosmetic, food-supplement, functionalization, hyaluronan applications, hyaluronan derivatives, hyaluronan synthases, hyaluronic acid, hyaluronidases, physico-chemical properties

## Abstract

Since its first isolation in 1934, hyaluronic acid (HA) has been studied across a variety of research areas. This unbranched glycosaminoglycan consisting of repeating disaccharide units of *N*-acetyl-d-glucosamine and d-glucuronic acid is almost ubiquitous in humans and in other vertebrates. HA is involved in many key processes, including cell signaling, wound reparation, tissue regeneration, morphogenesis, matrix organization and pathobiology, and has unique physico-chemical properties, such as biocompatibility, biodegradability, mucoadhesivity, hygroscopicity and viscoelasticity. For these reasons, exogenous HA has been investigated as a drug delivery system and treatment in cancer, ophthalmology, arthrology, pneumology, rhinology, urology, aesthetic medicine and cosmetics. To improve and customize its properties and applications, HA can be subjected to chemical modifications: conjugation and crosslinking. The present review gives an overview regarding HA, describing its history, physico-chemical, structural and hydrodynamic properties and biology (occurrence, biosynthesis (by hyaluronan synthases), degradation (by hyaluronidases and oxidative stress), roles, mechanisms of action and receptors). Furthermore, both conventional and recently emerging methods developed for the industrial production of HA and its chemical derivatization are presented. Finally, the medical, pharmaceutical and cosmetic applications of HA and its derivatives are reviewed, reporting examples of HA-based products that currently are on the market or are undergoing further investigations.

## 1. Introduction and Historical Background of HA

Research on hyaluronic acid (HA) has expanded over more than one century.

The first study that can be referred to regarding HA dates from 1880: the French scientist Portes observed that mucin from vitreous body was different from other mucoids in cornea and cartilage and called it “hyalomucine” [1]. Nevertheless, only in 1934, Meyer and Palmer isolated from bovine vitreous humor a new polysaccharide containing an amino sugar and a uronic acid and named it HA, from “hyaloid” (vitreous) and “uronic acid” [2]. During the 1930s and 1950s, HA was isolated also from human umbilical cord, rooster comb and streptococci [3,4]. 

The physico-chemical properties of HA were widely studied from the 1940s [5,6,7,8,9], and its chemical structure was solved in 1954 by Meyer and Weissmann [10]. During the second half of the Twentieth Century, the progressive understanding of HA’s biological roles [11,12,13] determined an increasing interest in its production and development as a medical product for a number of clinical applications. Hence, the extraction processes from animal tissues were progressively optimized, but still carried several problems of purification from unwanted contaminants (i.e., microorganisms, proteins). The first studies on HA production through bacterial fermentation and chemical synthesis were carried out before the 1970s [1]. 

The first pharmaceutical-grade HA was produced in 1979 by Balazs, who developed an efficient method to extract and purify the polymer from rooster combs and human umbilical cords [14]. Balazs’ procedure set the basis for the industrial production of HA [14]. Since the early 1980s, HA has been widely investigated as a raw material to develop intraocular lenses for implantation, becoming a major product in ophthalmology for its safety and protective effect on corneal endothelium [15,16,17,18,19,20,21,22]. Additionally, HA was found to be beneficial also for the treatment of joint [23,24,25,26,27] and skin diseases [28,29], for wound healing [30,31,32,33] and for soft tissue augmentation [34,35]. Since the late 1980s, HA has also been used to formulate drug delivery systems [36,37,38,39,40,41], and efforts continue still to today to develop HA-based vehicles to improve therapeutic efficacy [42,43,44,45]. During the 1990s and 2000s, particular attention was paid to identifying and characterizing the enzymes involved in HA metabolism, as well as developing bacterial fermentation techniques to produce HA with controlled size and polydispersity [1]. Nowadays, HA represents a key molecule in a variety of medical, pharmaceutical, nutritional and cosmetic applications. For this reason, HA is still widely studied to elucidate its biosynthetic pathways and molecular biology, to optimize its biotechnological production, to synthesize derivatives with improved properties and to optimize and implement its therapeutic and aesthetic uses [1,42,43,44,46,47,48,49,50,51,52,53,54,55,56,57,58]. 

Considering the great interest in HA from different fields, the fast growing number of studies and our interest in this topic, we decided to provide a comprehensive overview regarding HA and its potentialities, giving a concise update on the latest progress. As an example, a search on the most common public databases (i.e., Pubmed, Scopus, Isi Web of Science, ScienceDirect, Google Scholar, ResearchGate and Patent Data Base Questel) with the keyword “hyaluron*”, gave a total of 161,863 hits: 142,575 papers and 19,288 patents. This huge amount of data are continuously growing. Thus, with the aim to give a clearer picture about where researches and applications in the field are going, the present work starts with an update of HA’s physico-chemical, structural and hydrodynamic properties and proceeds with the discussion of HA biology: occurrence, biosynthesis (by hyaluronan synthases), degradation (by hyaluronidases and oxidative stress), roles, mechanisms of action and receptors. Furthermore, both conventional and recently-emerging methods developed for the industrial production of HA and its chemical derivatization are described. Finally, the medical, pharmaceutical, cosmetic and dietary applications of HA and its derivatives are reviewed, reporting examples of HA-based products that currently are on the market or are undergoing further investigations.

Literature search: we searched the Cochrane Controlled Trials Register (Central), Medline, EMBase and Cinahl from inception to November 2006 using truncated variations of preparation names including brand names combined with truncated variations of terms related to osteoarthritis, all as text. No methodologic filter for controlled clinical trials was applied (the exact search strategy is available from the authors). We entered relevant articles into the Science Citation Index to retrieve reports that have cited these articles, manually searched conference proceedings and textbooks, screened reference lists of all obtained articles and checked the proceedings of the U.S. Food and Drug Administration advisory panel related to relevant approval applications. Finally, we asked authors and content experts for relevant references and contacted manufacturers known to have conducted trials on viscosupplementation.

## 2. Physico-Chemical, Structural and Hydrodynamic Properties of HA

HA is a natural and unbranched polymer belonging to a group of heteropolysaccharides named glycosaminoglycans (GAGs), which are diffused in the epithelial, connective and nervous tissues of vertebrates [46,59,60]. All the GAGs (i.e., HA, chondroitin sulfate, dermatan sulfate, keratin sulfate, heparin sulfate and heparin) are characterized by the same basic structure consisting of disaccharide units of an amino sugar (*N*-acetyl-galactosamine or *N*-acetyl-glucosamine) and a uronic sugar (glucuronic acid, iduronic acid or galactose). However, HA differs as it is not sulfated and it is not synthesized by Golgi enzymes in association with proteins [46,59,60]. Indeed, HA is produced at the inner face of the plasma membrane without any covalent bond to a protein core. Additionally, HA can reach a very high molecular weight (HMW, 10^8^ Da), while the other GAGs are relatively smaller in size (<5 × 10^4^ Da, usually 1.5–2 × 10^4^ Da) [46,59,60].

**Figure 1:**
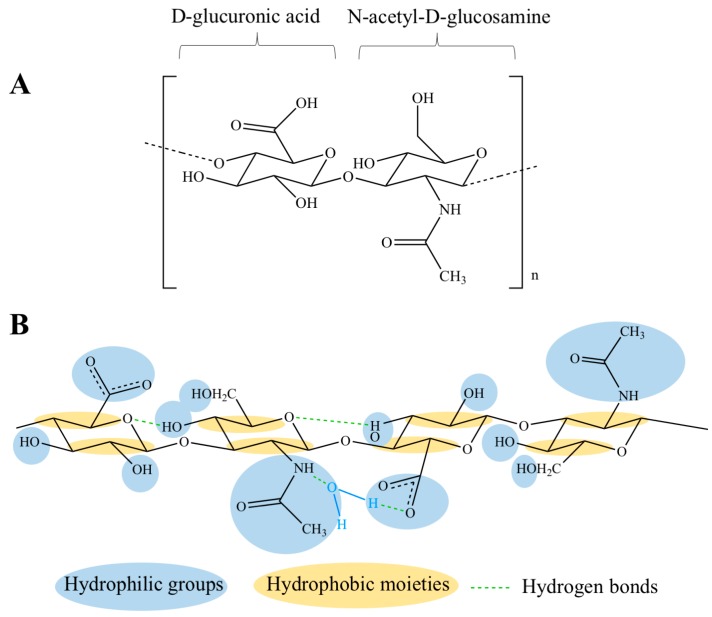
Chemical structures of HA disaccharide unit (**A**) and HA tetrasaccharide unit where the hydrophilic functional groups and the hydrophobic moieties are respectively evidenced in blue and yellow, while the hydrogen bonds are represented by green dashed lines (**B**).

The primary structure of HA is a linear chain containing repeating disaccharide units linked by ß-1,4-glycosidic bonds. Each disaccharide consists of *N*-acetyl-d-glucosamine and d-glucuronic acid connected by ß 1,3-glycosidic bonds (Figure 1) [10,61]. When both the monosaccharides are in the ß configuration, a very energetically-stable structure is formed, as each bulky functional group (hydroxyl, carboxyl, acetamido, anomeric carbon) is in the sterically-favorable equatorial position, while each small hydrogen atom occupies the less energetically-favorable axial position [62]. Thus, the free rotation around the glycosidic bonds of HA backbone is limited, resulting in a rigid conformation where hydrophobic patches (CH groups) are alternated with polar groups [63,64], which are linked by intra- and inter-molecular hydrogen bonds (H-bonds) (Figure 1) [65]. At physiological pH, each carboxyl group has an anionic charge, which can be balanced with a mobile cation such as Na^+^, K^+^, Ca^2+^ and Mg^2+^. Hence, in aqueous solution, HA is negatively charged and forms salts generally referred to as hyaluronan or hyaluronate [66,67], which are highly hydrophilic and, consequently, surrounded by water molecules. More precisely, as displayed in Figure 1, water molecules link HA carboxyl and acetamido groups with H-bonds that stabilize the secondary structure of the biopolymer, described as a single-strand left-handed helix with two disaccharide residues per turn (two-fold helix) [68]. In aqueous solution, HA two-fold helices form duplexes, i.e., a ß-sheet tertiary structure, due to hydrophobic interactions and inter-molecular H-bonds, which enable the aggregation of polymeric chains with the formation of an extended meshwork [64,65].

**Figure 2:**
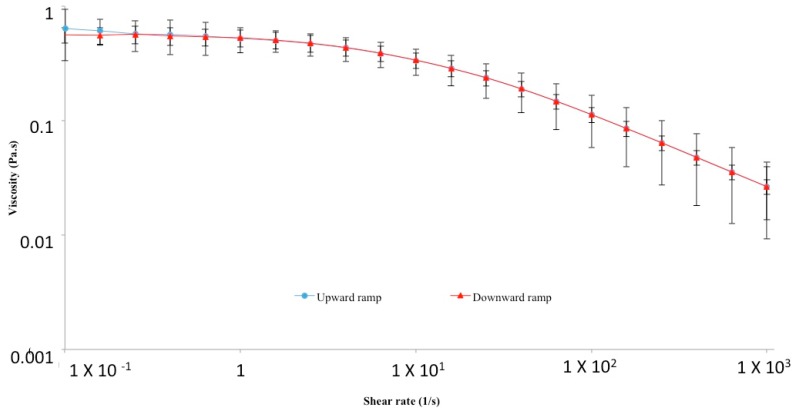
Shear-thinning and non-thixotropic behavior of 0.5% HA solution (2 MDa) analyzed using the rotational rheometer AR2000 (TA instruments, New Castle, DE, USA), connected to the Rheology Advantage software (Version V7.20) and equipped with an aluminum cone/plate geometry (diameter 40 mm, angle 2°, 64-μm truncation). The viscosity decreases in response to gradual increases of the shear rate over time (upward ramp), and then, the viscosity increases in response to gradual decreases of the shear rate over time (downward ramp). The initial viscosity is recovered through the same intermediate states of the breakdown process: the breakdown of the polymeric network is transient and reversible, and therefore, the original structure of HA is recovered.

The establishment of this network depends on HA molecular weight (MW) and concentration; for example, HMW native HA (>10^6^ Da) forms an extended network even at a very low concentration of 1 µg/mL [64,69]. With increasing MW and concentration, HA networks are strengthened, and consequently, HA solutions display progressively increased viscosity and viscoelasticity [70]. Since hyaluronan is a polyelectrolyte [71], its rheological properties in aqueous solutions are influenced also by ionic strength, pH and temperature [46,70,72]: as these factors increase, HA viscosity declines markedly, suggesting a weakening of the interactions among the polymer chains [73]. In particular, HA is highly sensitive to pH alterations: in acidic and alkaline environments, a critical balance between repulsive and attractive forces occurs [74], and when the pH is lower than four or higher than 11, HA is degraded by hydrolysis [75]. In alkaline conditions, this effect is more pronounced, due to the disruption of H bonds, which take part in the structural organization of HA chains [74,76,77]. Therefore, both the structural properties and the polyelectrolyte character of HA determine its rheological profile [65,73,78,79]. HA solutions are characterized by a non-Newtonian, shear-thinning and viscoelastic behavior. The shear-thinning (or pseudoplastic) profile of HA is due to the breakdown of the inter-molecular hydrogen bonds and hydrophobic interactions under increasing shear rates: HA chains deform and align in the streamlines of flow, and this results in a viscosity decrease [74,78] (Figure 2). Additionally, HA solutions are non-thixotropic: as the shear rate decreases and ends, they recover their original structure and viscosity proceeding through the same intermediate states of the breakdown process [73] (Figure 2). Hence, the breakdown of the polymeric network is transient and reversible. This unique rheological behavior is peculiar and extremely important, as it determines many physiological roles and pharmaceutical, medical, food and cosmetic applications of hyaluronan.

## 3. Biology of HA

### 3.1. HA Occurrence in Living Organism and Diffusion in the Human Body

Hyaluronan is widely diffused in nature: it is present in humans, animals, such as, rabbits, bovines, roosters, bacteria, such as *Streptococcus equi*, *Streptococcus zooepidermicus*, *Streptococcus equisimilis*, *Streptococcus pyogenes*, *Streptococcus uberis*, *Pasteurella multocida* [49,80,81,82], algae, such as the green algae *Chlorella* sp. infected by the *Chlorovirus* [49,83], yeasts, such as *Cryptococcus neoformans* [49], and mollusks [84]. However, it is not found in fungi, plants and insects [85].

In the human body, the total content of HA is about 15 g for a 70-kg adult [86]. HA is prevalently distributed around cells, where it forms a pericellular coating, and in the extracellular matrix (ECM) of connective tissues [61,82]. Approximately 50% of the total HA resides in the skin, both in the dermis and the epidermis [82]. Synovial joint fluid and eye vitreous body, being mainly composed of ECM, contain important amounts of hyaluronan: 3–4 mg/mL and 0.1 mg/mL (wet weight), respectively [61,82]. Moreover, HA is also abundant in the umbilical cord (4 mg/mL), where it represents the major component of Wharton’s jelly together with chondroitin sulfate [87,88]. The turnover of HA is fast (5 g/day) and is finely regulated through enzymatic synthesis and degradation [86].

### 3.2. HA Synthesis in the Human Body

**Figure 3:**
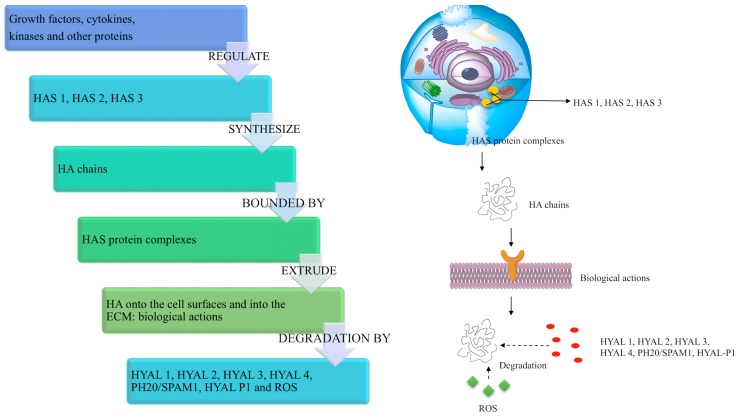
Schematic diagram showing HA key steps from its synthesis to its degradation.

In the human body, HA is synthesized as a free linear polymer by three transmembrane glycosyltransferase isoenzymes named hyaluronan synthases, HAS: HAS1, HAS2 and HAS3, whose catalytic sites are located on the inner face of the plasma membrane. HA growing chains are extruded onto the cell surface or into the ECM through the plasma membrane and HAS protein complexes [89,90] (Figure 3). The three HAS isoforms share the 50–71% of their amino acid sequences (55% HAS1/HAS2, 57% HAS1/HAS3, 71% HAS2/HAS3), and indeed, they are all characterized by seven membrane-spanning regions and a central cytoplasmic domain [50,86,89]. However, HAS gene sequences are located on different chromosomes (hCh19-HAS1, hCh8-HAS2 and hCh16-HAS3) [91,92], and the expression and the activity of HAS isoforms are controlled by growth factors, cytokines and other proteins such as kinases in different fashions, which appear cell and tissue specific [50,90,93,94]. Hence, the three HAS genes may respond differently to transcriptional signals: for example, in human fibroblasts like synoviocytes, transforming growth factor ß upregulates HAS1 expression, but downregulates HAS3 expression [95]. Moreover, HAS biochemical and synthetic properties are different: HAS1 is the least active isoenzyme and produces HMW hyaluronan (from 2 × 10^5^ to 2 × 10^6^ Da). HAS2 is more active and synthesizes HA chains greater than 2 × 10^6^ Da. It represents the main hyaluronan synthetic enzyme in normal adult cells, and its activity is finely regulated [96]. HAS2 also regulates the developmental and reparation processes of tissue growth, and it may be involved in inflammation, cancer, pulmonary fibrosis and keloid scarring [55,86,97,98,99]. HAS3 is the most active isoenzyme and produces HA molecules with MW lower than 3 × 10^5^ Da [60].

Dysregulation and misregulation of HAS genes’ expression result in abnormal production of HA and, therefore, in increased risk of pathological events, altered cell responses to injury and aberrant biological processes such as malignant transformation and metastasis [47,48,50,100].

Even if the exact regulation mechanisms and functions of each HAS isoenzyme have not been fully elucidated yet [96], all the aforementioned studies suggest that HAS are critical mediators of physiological and pathological processes, as they are involved in development, injury and disease.

### 3.3. HA Degradation in the Human Body

HA degradation in the human body is accomplished by two different mechanisms: one is specific, mediated by enzymes (hyaluronidases (HYAL)), while the other is nonspecific, determined by oxidative damage due to reactive oxygen species (ROS) (Figure 3). Together, HYAL and ROS locally degrade roughly 30% of the 15 g HA present in the human body. The remaining 70% is catabolized systemically: hyaluronan is mostly transported by the lymph to the lymph nodes, where it is internalized and catabolized by the endothelial cells of the lymphatic vessels. Additionally, a small part of HA is carried to the bloodstream and degraded by liver endothelial cells [50].

HYAL have a pivotal regulatory function in the metabolism of hyaluronan. These enzymes predominantly degrade HA, even if they are able to catabolize also chondroitin sulfate and chondroitin [101]. Randomly cleaving the β-*N*-acetyl-d-glucosaminidic linkages (β-1,4 glycosidic bonds) of HA chains, HYAL are classified as endoglycosidases. In the human genome, six HYAL gene sequences have been identified in two linked triplets: HYAL 1, HYAL 2, HYAL 3 genes, clustered on chromosome 3p21.3; HYAL-4 and PH20/SPAM1 genes, similarly located on chromosome 7p31.3, together with HYAL-P1 pseudogene [102]. HYAL have a consistent amino acid sequence in common: in particular, HYAL 1, HYAL 2, HYAL 3, HYAL 4 and PH20/SPAM1 share about 40% of their identity [101]. The expression of HYAL appears tissue specific. Nowadays, much is still unknown about HYAL activity, functions and posttranslational processing. HYAL-1, HYAL 2 and PH20/SPAM1 are the most characterized human HYAL. Both HYAL-1 and HYAL 2 have an optimal activity at acidic pH (≤4) [103,104] and are highly expressed in human somatic tissues [102]. HYAL 1 was the first human HYAL to be isolated: it was purified from serum (60 ng/mL) [105] and, successively, from urine [106]. HYAL 1 was found to regulate cell cycle progression and apoptosis: it is the main HYAL expressed in cancers, and therefore, it may regulate tumor growth and angiogenesis [107]. HYAL 1 works together with HYAL 2 to degrade HA, possibly according to the following mechanism, which is still the object of study. HYAL 2 is anchored on the external side of the cell surface: here, it cleaves into oligosaccharides (approximately 25 disaccharide units, 2 × 10^4^ Da) and the extracellular HMW HA (≥10^6^ Da), which is linked to its receptor cluster of differentiation-44 (CD44). These intermediate fragments are internalized, transported first to endosomes and then to lysosomes, where they are degraded into tetrasaccharide units (800 Da) by HYAL-1 [51]. Differently from HYAL-1 and HYAL-2, PH20/SPAM1 shows not only endoglycosidase activity both at acidic and neutral pH, but also a role in fertilization [108]. Hence, PH20/SPAM1 is unique among HYAL, as it behaves as a multifunctional enzyme.

HMW hyaluronan can also be naturally degraded in the organism by ROS, including superoxide, hydrogen peroxide, nitric oxide, peroxynitrite and hypohalous acids, which are massively produced during inflammatory responses, tissue injury and tumorigenesis [60,109]. The depolymerization of HA occurs through mechanisms of the reaction that are dependent on the ROS species, but always involve the scission of the glycosidic linkages [86,110]. Studies have shown that oxidation-related inflammatory processes, determining HA fragmentation, can increase the risk of injury in the airways and determine loss of viscosity in synovial fluid, with consequent cartilage degeneration, joint stiffness and pain [111,112,113]. ROS-induced degradation of HA might suggest why its antioxidant activity is one of its possible roles in reducing inflammation; however, so far, this biological function of HA has only been hypothesized, as it is not sufficiently supported by experimental data.

Due to these degradation mechanisms, which continuously occur in vivo, it has been estimated that the half-life of HA in the skin is about 24 h, in the eye 24–36 h, in the cartilage 1–3 weeks and in the vitreous humor 70 days [82].

### 3.4. Biological Roles of HA in Relation to Its MW

The equilibrium between HA synthesis and degradation plays a pivotal regulatory function in the human body, as it determines not only the amount, but also the MW of hyaluronan. Molecular mass and circumstances of synthesis/degradation are the key factors defining HA’s biological actions [50,51,100]. Indeed, high molecular weight (HMW) and low molecular weight (LMW) hyaluronan can even display opposite effects [51,60], and when they are simultaneously present in a specific tissue, they can exert actions different from the simple sum of those of their separate size-related effects [51].

Extracellular HMW HA (≥10^6^ Da) is anti-angiogenic, as it is able to inhibit endothelial cell growth [51,60,114]. Additionally, due its viscoelasticity, it acts as a lubricating agent in the synovial joint fluid, thus protecting the articular cartilage [115]. HMW HA has also important and beneficial roles in inflammation, tissue injury and repair, wound healing and immunosuppression: it binds fibrinogen and controls the recruitment of inflammatory cells, the levels of inflammatory cytokines and the migration of stem cells [60,93,114].

During some environmental and pathological conditions, such as asthma, pulmonary fibrosis and hypertension, chronic obstructive pulmonary disease and rheumatoid arthritis, HMW HA is cleaved into LMW HA (2 × 10^4^–10^6^ Da), which has been shown to possess pro-inflammatory and pro-angiogenic activities [51,100]. Indeed, LMW hyaluronan is able to stimulate the production of proinflammatory cytokines, chemokines and growth factors [51] and to promote ECM remodeling [50]. Moreover, LMW HA can also induce tumor progression, exerting its influence on cells [51,116] and provoking ECM remodeling.

Both anti- and pro-inflammatory properties have been displayed by oHA and HA fragments (≤2 × 10^4^ Da), depending on cell type and disease. Certain studies have shown that oHA are able to reduce Toll-like receptors (TLRs)-mediated inflammation [117], inhibit HA-CD44 activation of kinases [118] and retard the growth of tumors [119]. However, oHA have been also found to promote inflammation in synovial fibroblasts [120], stimulate cell adhesion [121] and enhance angiogenesis during wound healing [53].

Therefore, HA is clearly a key molecule involved in a number of physiological and pathological processes. However, despite the intensive studies carried out so far, still little is known about HA’s biological roles, the factors determining HA accumulation in transformed connective tissues and the consequent cancer progression, and much less is known about their dependence on hyaluronan molecular size and localization (intra- or extra-cellular). Further researches focusing on HA molecular biology and mechanisms of action are necessary to clarify all these aspects and may facilitate the development of novel HA-based therapies.

### 3.5. Mechanisms of Action of HA

HA performs its biological actions (Section 3.4.) according to two basic mechanisms: it can act as a passive structural molecule and as a signaling molecule. Both of these mechanisms of action have been shown to be size-dependent [51,86].

The passive mechanism is related to the physico-chemical properties of HMW HA. Due to its macromolecular size, marked hygroscopicity and viscoelasticity, HA is able to modulate tissue hydration, osmotic balance and the physical properties of ECM, structuring a hydrated and stable extracellular space where cells, collagen, elastin fibers and other ECM components are firmly maintained [59,86,88].

HA also acts as a signaling molecule by interacting with its binding proteins. Depending on HA MW, location and on cell-specific factors (receptor expression, signaling pathways and cell cycle), the binding between HA and its proteins determines opposite actions: pro- and anti-inflammatory activities, promotion and inhibition of cell migration, activation and blockage of cell division and differentiation. All the factors that determine HA activities as a signaling molecule could be related: MW may influence HA uptake by cells and may affect receptor affinity. Additionally, receptor complexes may cluster differently depending on HA MW [51].

HA binding proteins can be distinguished into HA-binding proteoglycans (extracellular or matrix hyaloadherins) and HA cell surface receptors (cellular hyaloadherins) [51]. HA has shown two different molecular mechanisms of interaction with its hyaloadherins. First, HA can interact in an autocrine fashion with its receptors on the same cell [60]. Second, it can behave as a paracrine substance, which binds its receptors on neighboring cells and thus activates different intracellular signal cascades. If HA has an HMW, a single chain can interact simultaneously with several cell surface receptors and can bind multiple proteoglycans. These structures, in turn, can aggregate with additional ECM proteins to form complexes, which can be linked to the cell surface through HA receptors [60,100]. Hence, HA acts as a scaffold that stabilizes the ECM structure not only through its passive structural action, but also through its active interaction with several extracellular hyaloadherins, such as aggrecan (prominent in the cartilage), neurocan and brevican (prominent in the central nervous system) and versican (present in different soft tissues) [60]. For these reasons, pericellular HA is involved in the preservation of the structure and functionality of connective tissues, as well as in their protection from environmental factors [88].

#### HA Cell Surface Receptors

**Figure 4:**
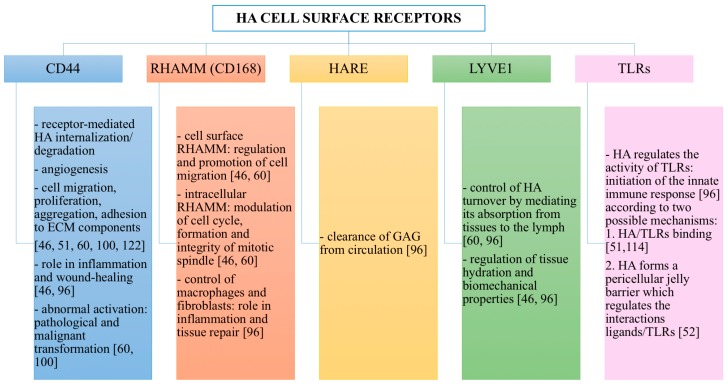
Summary of HA cell surface receptors and of the actions that they control when linked by HA.

HA interactions with its cell surface receptors mediate three biological processes: signal transduction, formation of pericellular coats and receptor-mediated internalization [60]. The present subsection describes HA cell surface receptors and the biological actions that they control when linked by HA (Figure 4).

The principal receptor for HA is CD44, which is a multifunctional transmembrane glycoprotein. It is expressed in many isoforms diffused in almost all human cell types. CD44 can interact not only with HA, but also with different growth factors, cytokines and extracellular matrix proteins as fibronectin [96]. CD44 intracellular domain interacts with cytoskeleton; hence, when its extracellular domain binds ECM hyaluronan, a link between the cytoskeletal structures and the biopolymer is created [46]. HA-CD44 interaction is involved in a variety of intracellular signaling pathways that control cell biological processes: receptor-mediated hyaluronan internalization/degradation, angiogenesis, cell migration, proliferation, aggregation and adhesion to ECM components [46,51,60,100,122]. Hence, CD44 plays a critical role in inflammation and wound healing [46,96]. However, abnormal activation of HA-CD44 signaling cascades, as well as overexpression and upregulation of CD44 (due to pro-inflammatory cytokines such as interleukin-1, and growth factors such as epidermal growth factors) can result into development of pathological lesions and malignant transformation [60,100]. Indeed, CD44 is overexpressed in many solid tumors, such as pancreatic, breast and lung cancer [54].

The receptor for HA-mediated cell motility (RHAMM) is also known as CD168, and it was the first isolated cellular hyaloadherin. It exists in several isoforms, which can be present not only in the cell membrane, but also in the cytoplasm and in the nucleus [96]. When liked by HA, cell surface RHAMM mediates and promotes cell migration, while intracellular RHAMM modulates the cell cycle, the formation and the integrity of mitotic spindle [46,60]. Interactions of HA with RHAMM play important roles in inflammation and tissue repair, by triggering a variety of signaling pathways and thus controlling cells such as macrophages and fibroblasts [96].

Hyaluronan receptor for endocytosis (HARE) was initially isolated from endothelial cells in the liver, lymph nodes and spleen and successively found also in endothelial cells of eye, brain, kidney and heart [96]. It is able to bind not only HA, but also other GAGs, with the exception of keratin sulfate, heparin sulfate and heparin. It is involved in the clearance of GAGs from circulation [96].

Furthermore, lymphatic vessel endothelial hyaluronan receptor 1 (LYVE1, a HA-binding protein expressed in lymph vascular endothelium and macrophages) controls HA turnover by mediating its adsorption from tissues to the lymph [60,96]. In this way, LYVE1 is involved in the regulation of tissue hydration and their biomechanical properties [46,96]. Additionally, LYVE1 forms complexes with growth factors, prostaglandins and other tissue mediators, which are implicated in the regulation of lymphangiogenesis and intercellular adhesion [46,96].

Finally, HA is involved in the regulation of the activity of TLRs that, recognizing bacterial lipopolysaccharides and lipopeptides, are able to initiate the innate immune response [96]. Two possible mechanisms have been proposed to explain how HA can influence TLRs. According to the first theory, LMW hyaluronan acts as an agonist for TLR2 and TLR4, thus provoking an inflammatory reaction [51,114]. On the contrary, according to the second theory, hyaluronan does not bind to TLRs, but it is able to regulate TLRs interactions with their ligands through the pericellular jelly barrier that it forms [52]. Indeed, in physiological conditions, HMW HA creates a dense and viscous protective coat around the cells, thus covering surface receptors such as TLRs and limiting their interactions with ligands. During inflammation, an imbalance between HA synthesis and degradation occurs, and this alters the thickness and the viscosity of HA pericellular barrier [52]. More precisely, HA is rapidly degraded due to pH reduction, ROS increase and the possible presence of pathogens producing HYAL [46,109]. Hence, HA MW decreases, reducing the polymer water binding ability and the thickness and the viscosity of its pericellular shield [52]. This results in an increased accessibility of the cell receptors to their ligands, in the initiation of the innate immune response and in the enhancement of the inflammatory reaction [52]. For this reason, HA can also be involved in the pathogenesis of diseases sustained by immunological processes [46].

## 4. Industrial Production of HA

The plethora of activities of a food-contained molecule has raised important interest for public health: the global market of HA was USD 7.2 billion in 2016, and it is expected to reach a value of USD 15.4 billion by 2025 [123]. Indeed, hyaluronan is gaining an exponentially growing interest for many pharmaceutical, medical, food and cosmetic applications, due to its important activities—anti-inflammatory, wound healing and immunosuppressive—and its numerous and incomparable biological and physico-chemical properties, such as biocompatibility, biodegradability, non-immunogenicity, mucoadhesivity, hygroscopicity, viscoelasticity and lubricity. Hence, there is a strong interest in optimizing HA production processes to obtain products that fulfill high quality standards and are characterized by great yield and accessible costs. Both the source and the purification process co-occur to determine the characteristics of the produced HA in terms of purity, MW, yield and cost [124,125]. Therefore, producing high quality HA with high yield and less costly methods represents one of the biggest challenges in the field of hyaluronan applied research.

The first production process applied at an industrial scale consisted of HA extraction from animal sources, such as bovine vitreous and rooster combs [46,49,124]. Despite the extraction protocols being improved over the years, this methodology was always hampered by several technical limitations, which led to the production of highly polydispersed HA (MW ≥ 10^6^ Da) with a low yield [1,46]. This was due to the polymer intrinsic polydispersity, its low concentration in tissues and its uncontrolled degradation caused by the endogenous HYAL and the harsh isolation conditions [46,49]. Additional disadvantages of animal-derived HA were represented by the risk of biological contamination—the presence of proteins, nucleic acids and viruses—and by the high purification costs [46,49,124]. Therefore, alternative methodologies for the industrial production of HA have been developed.

Currently, commercial hyaluronan is principally produced with biotechnology (microbial fermentation). Microorganism-derived HA is biocompatible with the human body because the HA structure is highly conserved among the different species [1,49]. Streptococci strains A and C were the first bacteria used for HA production, and nowadays, many commercial products are derived from *Streptococcus equi* (such as Restylane^®^ by Q-med AB and Juvederm^®^ by Allergan). Optimum bacterial culture conditions to obtain HMW HA (3.5–3.9 × 10^6^ Da) have been determined at 37 °C, pH 7, in the presence of lactose or sucrose [125,126]. Hyaluronan yield has been optimized up to 6–7 g/L, which is the upper technical limit of the process due to mass transfer limitation caused by the high viscosity of the fermentation broth [1]. As streptococci genera include several human pathogens, an accurate and expensive purification of the produced HA is necessary [46,49]. Hence, other microorganisms have been and are currently investigated to synthesize HA. An ideal microorganism for HA biosynthesis should be generally regarded as safe (GRAS), not secrete any toxins and be able to produce at least 10^6^ Da HA, as the polymer quality and market value increase with its purity and MW, which affect rheological and biological properties and define suitable applications [49,127]. Since the natural hyaluronan-producing organisms are mostly pathogenic, metabolic engineering currently represents an interesting opportunity to obtain HA from non-pathogenic, GRAS microorganisms. Endotoxin-free HA has already been synthesized by recombinant hosts including *Lactococcus lactis* [128], *Bacillus subtilis* [129], *Escherichia coli* [130] and *Corynebacterium glutamicum* [131]. However, up to now, there has been no heterologous bacterial host producing as much HA as the natural ones. Hence, there is an increasing effort to find an ideal bioreactor for HA production: in addition to bacteria, also eukaryotic organisms such as yeasts, like *Saccharomyces cerevisiae* [132] and *Pichia pastoris* [133], and plant cell cultures, like transformed tobacco-cultured cells [134], have been explored in the last few years.

Finally, to obtain HA of defined MW and narrow polydispersity, other approaches have been used. For example, to produce monodisperse oHA, chemoenzymatic synthesis has been performed [135]. This technique has successfully led to a product commercialized under the name Select HA™ (Hyalose LLC), characterized by a low polydispersity index value. Moreover, other studies have shown the possibility to prepare HA monodisperse fragments by controlling the degradation of HMW hyaluronan using different methods, including acidic, alkaline, ultrasonic and thermal degradation [110].

## 5. Synthetic Modifications of HA

HA has several interesting medical, pharmaceutical, food and cosmetic uses in its naturally-occurring linear form. However, chemical modifications of the HA structure represent a strategy to extend the possible applications of the polymer, obtaining better performing products that can satisfy specific demands and can be characterized by a longer half-life. During the design of novel synthetic derivatives, particular attention is paid to avoiding the loss of native HA properties such as biocompatibility, biodegradability and mucoadhesivity [46].

### 5.1. General Introduction of the Chemical Approaches to Modify HA

**Figure 5:**
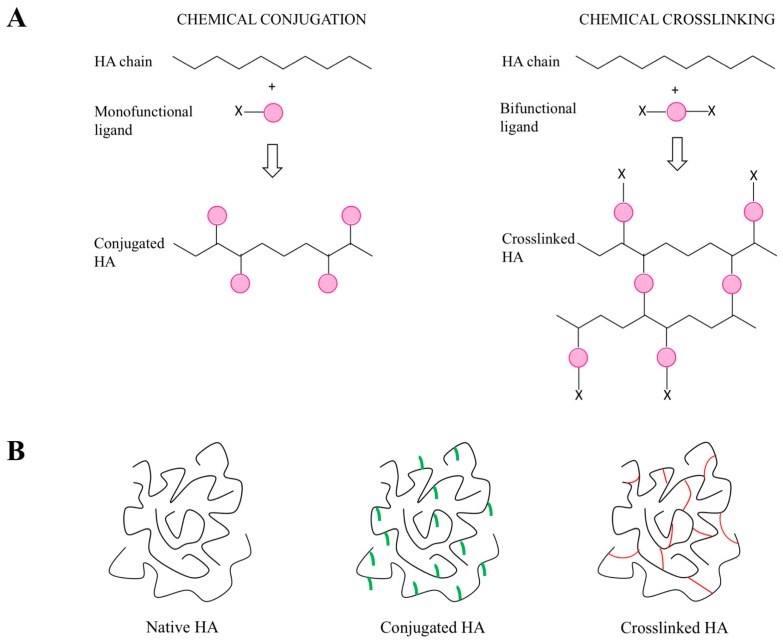
Chemical modifications of HA: conjugation and crosslinking (**A**). HA forms used for pharmaceutical, medical, food and cosmetic applications: native, conjugated and crosslinked (**B**).

HA chemical modifications mainly involve two functional sites of the biopolymer: the hydroxyl (probably the primary alcoholic function of the *N*-acetyl d glucosamine) and the carboxyl groups. Furthermore, synthetic modifications can be performed after the deacetylation of HA *N*-acetyl groups, a strategy that allows one to recover amino functionalities [136]. All these functional groups of HA can be modified through two techniques, which are based on the same chemical reactions, but lead to different products: conjugation and crosslinking (Figure 5). Conjugation consists of grafting a monofunctional molecule onto one HA chain by a single covalent bond, while crosslinking employs polyfunctional compounds to link together different chains of native or conjugated HA by two or more covalent bonds [136]. Crosslinked hyaluronan can be prepared from native HA (direct crosslinking) [56,58,137] or from its conjugates (see below). Conjugation and crosslinking are generally performed for different purposes. Conjugation permits crosslinking with a variety of molecules; to obtain carrier systems with improved drug delivery properties with respect to native HA; to develop pro-drugs by covalently linking active molecules to HA [136]. On the other hand, crosslinking is normally intended to improve the mechanical, rheological and swelling properties of HA and to reduce its degradation rate, in order to develop derivatives with a longer residence time in the site of application and greater release properties [58,138,139]. A recent trend is to conjugate and crosslink HA chains using bioactive molecules in order to develop derivatives with improved and customized activities [58] for a variety of applications in medicine, aesthetics and bioengineering, including cell and molecule delivery, tissue engineering and the development of scaffolds [46,56,58,140,141,142,143,144].

A number of synthetic approaches have been developed to produce conjugated or crosslinked hyaluronan [136].

Generally, HA is chemically modified in the liquid phase. Since it is hydrophilic, several reactions are performed in aqueous media also from its conjugates [145,146,147]: however, they are pH dependent and, therefore, require acidic or alkaline conditions, which if too strong, can determine HA degradation [75,145]. Other synthetic methods, involving the use of reagents sensitive to hydrolysis, are performed in anhydrous organic solvents such as dimethylsulfoxide (DMSO) [146] or dimethylformamide (DMF) [148]. These approaches necessarily introduce a preparation step to convert native HA into tetrabutylammonium (TBA) salt, soluble in organic ambient [148]: this increases the reaction time and cost, as well as the chances of HA chain fragmentation due to physico-chemical treatments. Additionally, when HA modifications take place in organic solvents, longer final purification processes are necessary [136]. The basic and classic chemistry that underlies the possible modifications of HA functional groups in the liquid phase is overviewed in the following Section 5.2, Section 5.3 and Section 5.4.

Generally, HA is chemically modified in the liquid phase. Since it is hydrophilic, several reactions are performed in aqueous media also from its conjugates [145,146,147]: however, they are pH dependent and, therefore, require acidic or alkaline conditions, which if too strong, can determine HA degradation [75,145]. Other synthetic methods, involving the use of reagents sensitive to hydrolysis, are performed in anhydrous organic solvents such as dimethylsulfoxide (DMSO) [146] or dimethylformamide (DMF) [148]. These approaches necessarily introduce a preparation step to convert native HA into tetrabutylammonium (TBA) salt, soluble in organic ambient [148]: this increases the reaction time and cost, as well as the chances of HA chain fragmentation due to physico-chemical treatments. Additionally, when HA modifications take place in organic solvents, longer final purification processes are necessary [136]. The basic and classic chemistry that underlies the possible modifications of HA functional groups in the liquid phase is overviewed in the following Section 5.2, Section 5.3 and Section 5.4.

Since HA derivatives of high quality and purity are necessary to develop injectable products, implantable scaffolds, drug delivery systems and 3D hydrogel matrices encapsulating living cells, techniques for efficient, low-cost and safe modification of HA are continuously being explored [46,147,149]. Hence, in the last few years, several efforts have been made to introduce one-pot reactions that preferably proceed in an aqueous environment, under mild and, possibly, environmentally-friendly conditions, without the use of toxic catalysts and reagents [147,149]. Additionally, alternative approaches to efficiently modify HA have been introduced: solvent-free methods, i.e., reactions in solid phase [57], “click chemistry” syntheses, which are simple and chemoselective, proceeding with fast kinetics in an aqueous environment, under mild conditions, leading to quantitative yields, without appreciable amounts of side products, i.e., the thiol-ene reaction [150], the Dies–Alder cycloaddition [151] and the azide-alkyne cycloaddition [152]; in situ crosslinking of functionalized HA through air oxidation [153]; photo-crosslinking of functionalized HA in the presence of photosensitizers [154,155].

### 5.2. Modification of HA Hydroxyl Groups

By modifying HA’s hydroxyl groups, the carboxyl groups remain unchanged, thus preserving HA’s natural recognition by its degradative enzymes [136]. Over the years, different derivatives of HA (ethers, hemiacetals, esters and carbamates) have been produced through reactions that occur between the polymeric hydroxyl groups and mono- or bi-functional agents.

Epoxides and bisepoxides like butanediol-diglycidyl ether (BDDE) [137], ethylene glycol-diglycidyl ether, polyglycerol polyglycidyl ether [156], epichlorohydrin and 1,2,7,8 diepoxyoctane [157] have been widely used to synthesize ether derivatives of hyaluronan in alkaline aqueous solution. Currently, HA-BDDE ether represents one of the most marketed HA derivative: it can be obtained through simple synthetic procedures in an aqueous environment, and it is degraded into non-cytotoxic fragments [136]. Other efficient methods to form ether derivatives of HA involve the use of divinyl sulfone (DVS) [158] or ethylene sulfide [159] in basic water.

Many studies showed that hemiacetal bonds can be formed between the hydroxyl groups of HA and glutaraldehyde in an acetone-water medium. Since glutaraldehyde is toxic, particular handling is required during the reaction and purification of the final product [160,161].

The hydroxyl groups of HA can be also esterified by reacting with octenyl succinic anhydride [162] or methacrylic anhydride [163] under alkaline conditions. Alternatively, HA can be converted into a DMSO-soluble salt, which can undergo esterification with activated compounds such as acyl-chloride carboxylates [164].

Finally, the activation of HA hydroxyl groups to cyanate esters, and the subsequent reaction in basic water with amines, allows one to synthesize carbamate derivatives with high degrees of substitution, in a reaction time of only 1 h [165].

### 5.3. Modification of HA Carboxyl Groups

Strategies for the derivatization of HA also involve esterification and amidation, which can be performed after the activation of the polymeric carboxyl groups using different reagents. By modifying HA’s carboxyl groups, derivatives more stable to HYAL degradation can be synthesized: hence, if a drug is conjugated on the carboxyl groups of HA, a slow drug release may occur [136].

Esterification can be performed by alkylation of HA carboxyl groups using alkyl halides [166] or tosylate activation [167]. Moreover, HA esters can be synthesized using diazomethane as the activator of the carboxyl groups [168]. All these reactions proceed in DMSO from the TBA salt of HA. Alternatively, HA can undergo esterification also in water using epoxides such as glycidyl methacrylate and excess trimethylamine as a catalyst [169]. The conversion of HA carboxyl groups into less hydrophilic esters represents a strategy to decrease the water solubility of HA, with the aim to reduce its susceptibility to HYAL degradation and enhance its in situ permanence time [46]. A well-known biopolymer synthesized to this end is HA benzyl ester (HYAFF 11), the properties of which are finely regulated by its degree of functionalization [36,170].

Amidation represents a further approach to modify HA: over the years, several synthetic procedures have been developed. However, some of these present important drawbacks: for example, Ugi condensation (useful to crosslink HA chains through diamide linkages) requires a strongly acidic pH (3), the use of formaldehyde, which is carcinogenic, and cyclohexyl isocyanide, which determines a pending undesired cyclohexyl group in the final product [145,160]. HA amidation with 1,1′-carbonyldiimidazole [171] or 2-chloro-1-methylpyridinium iodide [148] as activating agents are performed in DMSO and DMF, respectively: hence, HA conversion into TBA salt and longer purification steps are needed. On the contrary, other methods are based on reaction conditions that meet the modification requirements for HA. Particularly efficient is the activation of HA carboxyl groups by carbodiimide (i.e., *N*-(3-dimethylaminopropyl)-*N*′-ethylcarbodiimide hydrochloride) (EDC) and co-activators such are *N*-hydroxysuccinimide (NHS) or 1-hydroxybenzotriazole in water: proceeding under mild conditions, this reaction does not lead to HA chains’ cleavage, and it is suitable also for the derivatization with biopolymers easily susceptible to denaturation, such as protein or peptides [146,172,173]. Another promising method to synthesize HA derivatives with high grafting yields, in mild conditions, is based on triazine-activated amidation, typically performed with 2-chloro-dimethoxy-1,3,5-triazine [174] or (4-(4,6-dimethoxy-1,3,5-triazin-2-yl)-4-methylmorpholinium (DMTMM) [175]. A recent study made a systematic comparison of EDC/NHS and DMTMM activation chemistry for modifying HA via amide formation in water [175]. The results showed that DMTMM is more efficient than EDC/NHS for ligation of amines to HA and does not require accurate pH control during the reaction to be effective [175]. Using these mild conditions of amidation, it is possible to synthesize highly hydrophilic and biocompatible derivatives, such as urea-crosslinked HA, which has already shown interesting applications in the ophthalmic and aesthetics field [56,58].

### 5.4. Modification of HA N-Acetyl Groups

The deacetylation of the *N*-acetyl groups of HA recovers amino functionalities, which can then react with activated acids using the same amidation methods described above. However, this approach is not frequently used to synthesize HA derivatives for two main reasons: first of all, even the mildest deacetylation techniques have been shown to induce chain fragmentation [146,171,176]. Moreover, the deacetylation is a strong structural modification, which could importantly change the unique biological properties typical of native HA: indeed, it has been recently found that it reduces the interactions with the receptor CD44 [177].

## 6. Applications of HA and Its Derivatives

**Figure 6:**
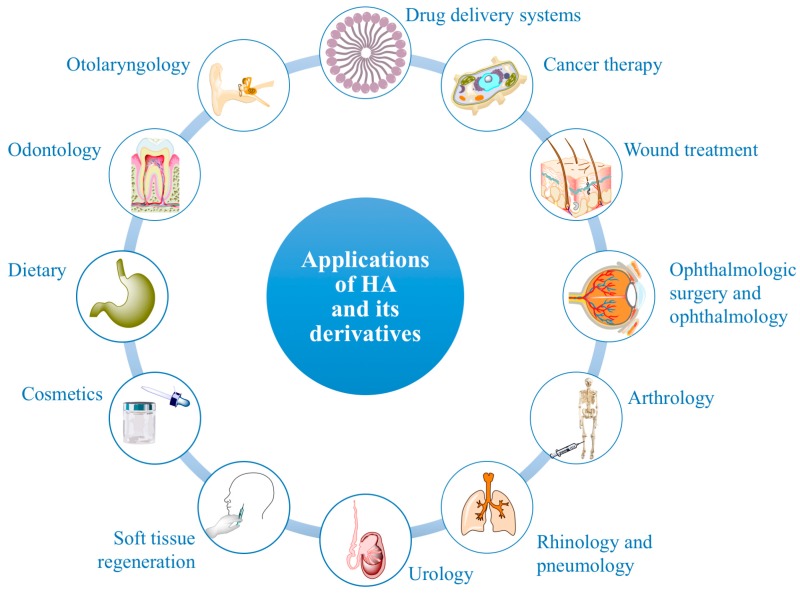
Medical, pharmaceutical, cosmetic and dietary applications of HA and its derivatives.

Due to their unique biological and physico-chemical properties and to their safety profile, native HA and many of its derivatives represent interesting biomaterials for a variety of medical, pharmaceutical, food and cosmetic applications (Figure 6). Some HA-based products are already on the market and/or have already a consolidated clinical practice, while others are currently undergoing further investigations to confirm their effectiveness. Since the literature concerning HA derivatives and their applications is very extensive, only some examples are reported hereafter.

### 6.1. Drug Delivery Systems

HA and its derivatives of synthesis represent useful and emerging tools to improve drug delivery. They have been used alone or in combination with other substances to develop pro-drugs, surface-modified liposomes, nanoparticles, microparticles, hydrogel and other drug carriers. All these delivery systems are undergoing a continuous optimization as they are the object of intensive research. However, the industrialization and extensive clinical application of HA and its derivatives as drug carriers still have a long way to go, as many scientific studies are only at an in vitro experimental stage.

The conjugation of active ingredients to HA is intended to develop pro-drugs with improved physico-chemical properties, stability and therapeutic efficacy compared to free drugs. Considering that hyaluronan has several biological functions, HA-drug conjugates can exert their activities as such. Alternatively, their therapeutic actions are accomplished when the drugs are released, i.e., when the covalent bonds, which link drugs and HA, are broken down in the organism, ideally at the specific target sites. A variety of active ingredients can be conjugated to HA for topical or systemic uses. For example, HA can be conjugated with antidiabetic peptides such as exendin 4: this derivative shows a prolonged half-life, a protracted hypoglycemic effect and improved insulinotropic activity compared to the free exendin 4 in type 2 diabetic mice [178]. However, since HA has a short half-life in the blood, the majority of HA-drug conjugates have been developed for local, i.e., intraarticular, intratumoral, subcutaneous, intravesical and intraperitoneal, rather than systemic administration [179]. For instance, a variety of anti-inflammatory drugs, including hydrocortisone, prednisone, prednisolone and dexamethasone, have been conjugated to HA and investigated for intraarticular therapy of arthritis [166]. Furthermore, a recent study has displayed the potential of an emulsion containing a novel HA-P40 conjugate to treat a mouse model of dermatitis induced by oxazolone [180]. P40 is a particulate fragment isolated from *Corynebacterium granulosum* (actually known as *Propionibacterium acnes*), which has immunomodulatory, antibacterial, antiviral and antitumor properties. The conjugation of P40 to HA successfully prevents its systemic absorption and, therefore, improves its topical therapeutic effect [180]. Other research has shown that conjugation of curcumin with HA represents a strategy to enhance the water solubility and stability of curcumin [181]. Moreover, the HA-curcumin conjugate displays improved healing properties compared to free curcumin and free HA, both in vitro (wound model of human keratinocytes) and in vivo (wound model of diabetic mouse). Hence, HA-conjugated curcumin may be useful to treat diabetic wounds [182]. Finally, the most promising and thoroughly studied conjugations of active ingredients to HA involve antitumoral agents, which can be strategically carried to malignant cells by hyaluronan, as explained in Section 6.2 [179,183].

HA can also be conjugated to phospholipids in order to develop surface-modified liposomes: the chemical modification can be performed prior to liposome formulation [184] or after, on the outside shell [185,186]. Moreover, HA can also be non-covalently linked on the liposome surface: indeed, liposomes can be covered by HA through ionic interaction mechanism [187,188] or the simple lipid film hydration technique [189]. HA-modified liposomes appear to be promising carriers, as they have been shown to enhance the stability of drugs in the bloodstream, prolong their half-life, reduce their systemic toxicity [186], enhance their tissue permeability, sustain their prolonged release [189] and ameliorate their therapeutic effects through synergistic actions [190]. HA-coated liposomes could improve the safety and the efficacy of antitumoral therapies: they appear proficient in mediating site-specific delivery of siRNA [191] and anticancer drugs such as doxorubicin [185], gemcitabine [186], imatinib mesylate [188] and docetaxel [184], via CD44 cell receptors. Additionally, HA-surface-modified liposomes have been investigated as delivery systems also in ophthalmology [189], pneumology [190] and topical treatment of wounds and burns [40].

Another promising type of drug delivery system, which can be formulated with HA and its derivatives of synthesis, is represented by nanoparticles. Hyaluronan can be a constituent element of nanoparticles [192,193], but can also be used to cover nanoparticles, in order to improve the targeting efficiency and the therapeutic action of the encapsulated drugs [194]. HA-nanoparticles are being investigated for a number of administration routes and customized applications: for example, HA-Flt1 peptide conjugate nanoparticles might represent a next-generation pulmonary delivery carrier for dexamethasone in the management of asthma [193], while chitosan nanoparticles coated with HA and containing betamethasone valerate have displayed a great potential for the topical treatment of atopic dermatitis [194]. Hyaluronan nanoparticles included in polymeric films have shown potential as innovative therapeutic system for the prolonged release of vitamin E for the management of skin wounds [195]. Moreover, the HA-poly(*N*-isopropylacrylamide) conjugate appears to be a promising candidate to treat osteoarthritis: once injected subcutaneously or intra-articularly, it spontaneously forms biocompatible nanoparticles able to control inflammation with a long-lasting action [192]. Finally, other HA surface-modified nanoparticles have been investigated for cancer-targeted therapies [196,197,198].

Additionally, over the years, HA microspheres and microparticles have been explored as formulations to improve the biomucoadhesive property and the drug release profile and to ameliorate the texturing feeling in the case of dermal formulations. For example, it has been shown that spray-dried HA microspheres allow favorable ofloxacin delivery to the lung via inhalation, determining a superior pharmacological effect compared to free ofloxacin and to other routes of ofloxacin administration [199]. Similarly, inhaled HA microparticles have displayed prolonged pulmonary retention of salbutamol sulfate and reduced systemic exposure and side effects in a rat model [200]. HA microspheres have been evaluated also as possible materials for bone supplementation: indeed, they could be introduced in mineral bone cements to extend the release of active compounds [201]. A recent work has shown the potential of caffeine-loaded HA microparticles dispersed in a lecithin organogel as a dermal formulation for the long-term treatment of cellulite: this drug delivery system is not only effective at repairing cellulite tissue damage, but has also an intrinsic moisturizing action [202]. Besides the microparticles prepared from native HA, the scientific literature also describes microspheres formulated from HA derivatives of synthesis such as hyaluronan benzyl esters [203] and DVS-crosslinked hyaluronan [138] as topical drug delivery systems.

Finally, hydrogels prepared from linear HA and its chemical derivatives are 3D polymeric networks, which can be well-suited systems for topical delivery of cells [204] and many active ingredients, such as anti-inflammatories [44], anti-bacterials [205], antibodies and proteins in general [42]. To implement the mechanical and release properties, HA hydrogels can incorporate thermoresponsive polymers [42] or other drug carriers such as liposomes [43,44]. Up to now, HA hydrogels have shown a great potential for intraocular [42,204], intratympanic [44], intraarticular [206] and dermal delivery [207]. A topical 2.5% HA hydrogel containing 3% diclofenac has been commercialized for the treatment of actinic keratosis under the tradename of Solaraze^®^ (Pharmaderm) in Europe, USA and Canada [207].

### 6.2. Cancer Therapy

It has been shown that the receptor CD44 is overexpressed in a variety of tumor cells, which consequently, display an increase of HA binding and internalization [46,54,208]. Hence, the receptor CD44 has been identified as a potential target in cancer therapy, and hyaluronan, its primary ligand, has been recognized as a powerful tool to develop targeted therapies [54,198]. Many research works have shown that HA can act as a drug carrier and targeting agent at the same time, under the form of polymer-antitumoral conjugates or delivery systems encapsulating anticancer drugs [179]. Additionally, hyaluronan can be employed to design surface-modified nanoparticles [196,197,198] or liposomes [184,185,186,187,188]. After CD44 receptor-mediated cell internalization, all these HA derivatives are hydrolyzed by intracellular enzymes, and therefore, drugs are released inside the cancer target cells [179]. This should improve the pharmacokinetic profile and the delivery process of many anticancer drugs, overcoming the limitations that reduce their clinical potential, such as low solubility, short in vivo half-life, lack of discrimination between healthy and malignant tissues, consequent off-target accumulation and side effects [54,179]. Up to now, there are no HA-antitumoral conjugates and anticancer loaded carriers of HA on the market; however, the promising results of many research works and clinical trials outline their potential and encourage further studies [54,179]. For example, it has been shown that HA-modified polycaprolactone nanoparticles encapsulating naringenin enhance drug uptake by cancer cells in vitro and inhibit tumor growth in rat with urethane-induced lung cancer [198]. Additionally, HA-coated chitosan nanoparticles have been found to promote 5-fluorouracil delivery into tumor cells that overexpress the CD44 receptor [196]. Further studies have displayed that a novel unsaturated derivative of HA [209] and different types of HA-paclitaxel conjugates [179,183] have a great potential as anticancer therapies.

### 6.3. Wound Treatment

As previously explained (Section 3.4.), endogenous HA sustains wound healing and re-epithelialization processes thanks to several actions including the promotion of fibroblast proliferation, migration and adhesion to the wound site, as well as the stimulation of collagen production [210]. For this reason, HA is used in topical formulations (such as Connettivina^®^ by Fidia) to treat skin irritations and wounds such as abrasions, post-surgical incisions, metabolic and vascular ulcers and burns [82,210,211,212,213]. Currently, HA derivatives [173,182,214,215] and HA-based wound dressings, films or hydrogels enriched with other therapeutic agents [216,217] are being evaluated in order to understand if the cicatrization process could be further enhanced. The wound healing properties of HA and its derivatives are being explored not only in dermatology, but also in other medical fields such as ophthalmology [56,218], otolaryngology [142], rhinology [219] and odontology [220].

### 6.4. Ophthalmologic Surgery and Ophthalmology

HA is a natural component of the human eye: it has been found in vitreous body, lacrimal gland, corneal epithelium and conjunctiva and tear fluid [56]. Therefore, ophthalmic products based on HA are fully biocompatible and do not trigger foreign body reactions [136].

HA solutions are the most used viscosurgical devices to protect and lubricate the delicate eye tissues, replace lost vitreous fluid and provide space for manipulation during ophthalmic interventions [221]. Indeed, the viscosity of HA permits keeping the tissues in place, reducing the risk of displacement, which can potentially compromise both the surgery and the repairing process [46]. The first ophthalmic viscosurgical device containing HA was approved by the FDA in 1980 and is still marketed under the trademark Healon^®^ (Abbott).

Moreover, HA is the active ingredient of many eye drops, such as DropStar^®^ by Bracco and Lubristil^®^ by Eyelab, which, hydrating the ocular surface and improving the quality of vision, are the mainstay to treat diseases such as dry eye syndrome and are useful at increasing the comfortability of contact lenses [56,222,223]. Many studies have proven the safety and the efficacy of native HA solutions as artificial tears [222,224,225,226]. More recently, also novel derivatives of hyaluronan with improved mechanical and biological properties are being investigated to formulate eye drops with enhanced ocular residence times. For example, promising preliminary results have been obtained with solutions of HA-cysteine ethyl ester [227] and urea-crosslinked HA (HA-CL) [56].

### 6.5. Arthrology

HA is one of the major lubricating agents of the ECM of synovial joint fluid: due to its viscoelasticity, it absorbs mechanical impacts and avoids friction between the bone-ends [61,82,115]. When the synovial fluid is reduced or inflamed, and the HA level decreases, disorders such as rheumatoid arthritis and osteoarthritis occur. Viscosupplementation represents an approach to treat and slow down the progression of these conditions: intraarticular injections of HMW HA, such as Supartz FX^®^ by Bioventus and Hyalgan^®^ by Fidia, allow maximizing the topical effect and the reduction of pain, as well as minimizing systemic adverse effects [140]. Locally-injected HA has been shown to provide long-term clinical benefits, suggesting that it acts with more than one mechanism [140,228], as the restoration of synovial fluid viscoelasticity is only temporary because HA is degraded within 24 h [229]. Hence, the therapeutic effect of HA intra-articular injection appears prevalently due to biological activities: induction of the synthesis of new HA in synovial cells, stimulation of chondrocyte proliferation and resulting reduction of cartilage degradation [140,228,230]. In order to increase the half-life after injection and, consequently, the therapeutic efficacy, crosslinked HA derivatives have been investigated and introduced on the market (such as Synvisc^®^ by Genzyme) as viscosupplementation agents [140,231].

### 6.6. Rhinology and Pneumology

Endogenous HMW HA plays a pivotal role in the homeostasis of the upper and the lower airways: it is an important component of the normal airway secretions, exerts anti-inflammatory and anti-angiogenic actions, promotes cell survival and mucociliary clearance, organizes extracellular matrix, stabilizes connective tissues, sustains healing processes and regulates tissues hydration [144,232,233,234]. Hence, exogenous HMW HA represents a promising therapeutic agent for the treatment of nasal and lung diseases that involve inflammation, oxidative stress and epithelial remodeling, such as allergic and non-allergic rhinitis, asthma, chronic obstructive pulmonary disease and cystic fibrosis [143,233,235,236,237,238]. Examples of marketed formulations containing HA to treat respiratory diseases are Ialoclean^®^ (Farma-Derma), a nasal spray to treat nasal dryness and rhinitis and to promote nasal wound healing, Hyaneb^®^ (Chiesi Farmaceutici), a hypertonic saline solution containing HA to hydrate and reduce mucus viscosity in cystic fibrosis patients [239], and Yabro^®^ (Ibsa Farmaceutici), a high viscosity nebulizer solution of HA to treat bronchial hyper-reactivity [143].

### 6.7. Urology

Recently, the possible therapeutic uses of HA in urology have been explored. Preliminary evidence has shown that intravesical HA, administered alone or in combination with chondroitin sulfate or alpha blockers, could be able to reduce the recurrence of urinary tract infections such as bacterial cystitis, to alleviate the symptoms of these diseases and to protect the mucosa of urinary bladder [240,241]. However, further clinical studies are necessary to confirm the effectiveness of HA treatment in urology.

### 6.8. Soft Tissue Regeneration

HA skin content decreases with aging, and the most visible effects are the loss of facial skin hydration, elasticity and volume, which are responsible for wrinkles [88]. Over the last few years, HA has been widely used as a biomaterial to develop dermal fillers (DFs), which are class III medical devices that, injected into or under the skin, restore lost volumes and correct facial imperfections such as wrinkles or scars [58]. Being characterized by most of the properties that an ideal DF should have—biocompatibility, biodegradability, viscoelasticity, safety, versatility—HA DFs have become the most popular agents for viscoaugmentation, i.e., for soft tissue contouring and volumizing [58]. Indeed, according to data from the American Society of Plastic Surgeons (ASPS), in 2017, out of a total of 2,691,265 treatments with soft tissue fillers, 2,091,476 were performed with HA DFs [242]. One of the reasons for this success resides in the reversibility of the HA DF effect: they correct wrinkles in a reversible manner, as a hypothetical medical error or complication can be remedied through the injection of HYAL (Vitrase^®^, ISTA Pharmaceuticals; Hylenex^®^, Halozyme Therapeutics) [58]. The duration of the corrective effect of HA DFs varies between three and 24 months, depending prevalently on HA concentration, crosslinking (degree and type), the treated area and the individual [58,243]. For example, Hylaform^®^ (Genzyme Biosurgery) contains 4.5–6 mg/mL HA crosslinked with DVS (20% degree), and its effect lasts 3–4 months, while the Juvederm^®^ DFs family (Allergan) contains 18–30 mg/mL HA crosslinked with 9–11% BDDE, and its effect lasts 6–24 months [58]. Finally, DFs that combine BTX-A and HA have been developed to ensure an optimal correction even in patients with extremely deep wrinkles [58].

### 6.9. Cosmetics

HA represents a moisturizing active ingredient widely used in cosmetic formulations (gels, emulsions or serums) to restore the physiological microenvironment typical of youthful skin. HA-based cosmetics such as Fillerina^®^ (Labo Cosprophar Suisse) claims to restore skin hydration and elasticity: this is reported to exert an anti-wrinkle effect, although no rigorous scientific proof is able to fully substantiate this claim [82,141,244]. It has to be considered that HA’s hydrating effect largely depends on its MW, and its longevity depends on HA stability to hyaluronidases. Indeed, HMW HA mainly works as a film-forming polymer: it reduces water evaporation, with an occlusive-like action. On the other hand, medium MW and LMW HA mainly work by binding moisture from the environment, do to their high hygroscopicity [141,244]. In some cases, this capacity may reverse HA’s expected hydrating activity as at a high concentration, HA may even extract humidity from the skin. Furthermore, also sunscreens containing hyaluronan may contribute to maintaining a youthful skin, protecting it against the harmful effects of ultraviolet irradiation, due to the possible free radical scavenging properties of HA [245,246]. The same capability has been demonstrated by dietary intake of HA (Section 6.10).

### 6.10. Dietary

HA can also represent an interesting ingredient in enriched food and food supplements: it has gained the unofficial designation as a nutri-cosmetic because of its capability to improve skin appearance [247]. For a long time, the fact that HA can cross in its “intact” form the intestinal barrier has been debated; recently, a few studies have appeared in the literature to highlight this question. Kimura et al. have evaluated the degradation and absorption of HA (300 KDa and 2 KDa) after oral ingestion in rats, demonstrating intestinal degradation to oligosaccharides, which are subsequently absorbed in the large intestine, translocated into the blood and distributed in the skin [248]. Orally-ingested LMW HA has shown the opposite effects: some studies have reported inflammatory properties with the activation of the immune response [249], while other research has highlighted the efficacy in reducing knee joint pain without inflammation [250]. Nowadays, the mechanisms at the base of these different actions of LMW ingested HA remain still unclear. In another study, the absorption, distribution and excretion of HMW-labeled (1 MDa) HA were evaluated after oral administration in rats and dogs: for the first time, it was shown that dietary HMW HA can be distributed to connective tissues [251]. In particular, these reproducible results suggest that orally-administered HMW HA may reach joints, bones and skin, even if in small amounts [251], thus highlighting that a rationale may exist in the use of HA-based food supplements designed for joint and skin health.

In these regards, several studies have reported on the safety of HA as a food supplement, confirming its possible use as a food ingredient itself. HA is marketed as a food supplement in the USA, Canada, Europe and Asia (particularly in Korea and Japan) with some difference in the suggested use: to treat joint pain in the USA and in Europe; to treat wrinkles and to moisturize in Japan, even if the involvement of this polymer in the skin moisture retention effect needs to be further elucidated in the future [252]. In the present review, we focused our attention prevalently on studies that exclude complex mixtures, in order to evidence HA’s effects alone; however, several research works describe the use of HA as a food ingredient in enriched extracts or mixtures with collagen and other supplements. For example, in a preliminary double-blind, controlled, randomized, parallel trial over 12 weeks, rooster comb extract was added to low fat yoghurt, which was given to mild knee pain patients (n: 40), resulting in significative improvement in muscle strength in men [253]. In another recent clinical study, an oral HA preparation diluted in a cascade fermented organic whole food concentrate supplemented with biotin, vitamin C, copper and zinc (Regulatpro^®^ Hyaluron, Dr. Niedermaier^®^) led to a significant cutaneous antiaging effect in twenty female subjects after 40 days of daily consumption [254]. From such mixtures, it is difficult to elucidate HA’s specific contribution.

Regarding supplementation with HA alone, some studies have demonstrated a direct correlation between ingestion and body effects. In a recent review, Kawada et al. underlined a number of studies in support of the contribution of ingested HA to hydrate skin, thus improving the quality of life for people who suffer from skin dryness induced by UV, smoking and pollutants, responsible for cutaneous reduction of HA [255]. Indeed, the review of Kawada and coworkers reported that, in different randomized, double-blind, placebo-controlled trials, the amounts of HA ranging from 37.52–240 mg per day, ingested in a period comprised between four and six weeks, significantly improved cutaneous moistness [255]. These results, as always happens with biopolymers as food supplements, are difficult to correlate with quantitative effects, as polymer sources and MW always vary. However, qualitatively, the correlation between HA consumption and the decrease of skin dryness is evident. The authors suggest that partially-digested HA, regardless of its MW, is adsorbed in the gut, while intact HA is absorbed by the lymphatic system; both are distributed to the skin, where they can work as inducers of fibroblast proliferation and endogenous HA synthesis [255]. In a more recent study, the same authors have investigated, in a double blind, placebo controlled, randomized study of 61 subjects with dry skin, the effects of HA (120 mg/day) of two different MW (800 KDa and 300 KDa) over six weeks [256]. Both the HAs were effective, but the 300-KDa group showed the best improvements in skin dryness and moisture content [256]. Again, Kawada et al., in an experiment on hairless mice, demonstrated that the oral administration of 200 mg/kg body weight per day of two different MW HA (300 KDa and less than 10 KDa) for six weeks reduced epidermal thickness and improved skin hydration upon UV irradiation [257]. The effect was stronger for the less than 10-KDa HA [257]. The authors also demonstrated that the less than 10 KDa orally-administered HA also stimulated HAS2 expression, thus highlighting an overall role of LMW HA in the prevention of skin photoageing with different mechanisms [257]. Thus, combining HA oral treatment with HA topical and injective administration could be very successful in the control of skin ageing. A further study suggesting that orally-administered HA can migrate into the skin of rats, thus possibly reducing skin dryness, was conducted by Oe et al., who demonstrated that about 90% of the ingested HA was absorbed from the digestive tract and was used as an energy source or a structural component [252].

Dietary HA can be beneficial not only for skin, but also for joints, as evidenced by a number of randomized, double-blinded, placebo-controlled studies relative to the treatment of knee pain, relief of synovial effusion or inflammation and improvement of muscular knee strength [258].

### 6.11. 3D Cell Culture Models

HA and its synthetic derivatives can be used as 3D scaffold structures, which represent physical support systems for in vitro cell culture [259]. Indeed, 3D tissue models can be obtained by culturing cells on pre-fabricated polymeric scaffolds or matrices, designed to simulate the in vivo ECM [259]. Cells attach, migrate and fill the interstices within the scaffold to form 3D cultures [259]. 3D scaffolds can be promising also for in vivo tissue regeneration, reproducing the natural physical and structural environment of living tissue [259]. An example of a cell substrate suitable for a 3D environment, for both in vitro and in vivo research, is represented by HyStem^®^ Hydrogels (ESI·BIO^TM^).

For example, HA derivatives characterized by aldehyde and hydrazide groups have been used to develop a biomimetic, 3D culture system for poorly-adherent metastatic prostate cancer cells, employed as an in vitro platform to test the efficacy of anticancer drugs [260]. The hyaluronan-3D cell culture system provided a useful interesting alternative to study antineoplastic drugs, with results superior compared to those from conventional 2D monolayers [260]. Another work showed that methacrylated HA is useful to develop in vitro 3D culture models to assess glial scarring in a robust and repeatable way, in order to evaluate, for example, the foreign body response to implants such as electrodes in the central nervous system [261]. Additionally, a recent study highlighted the importance of ink formulation and crosslinking on the printing of stable structures: a dual-crosslinking HA system was evaluated as printable hydrogel ink in biomedicine [262]. It encompassed shear-thinning and self-healing properties through guest-host bonding, and showed an improved cell adhesion after further functionalization (i.e., peptides) [262].

## 7. Conclusions, Future Trends and Perspectives

**Figure 7:**
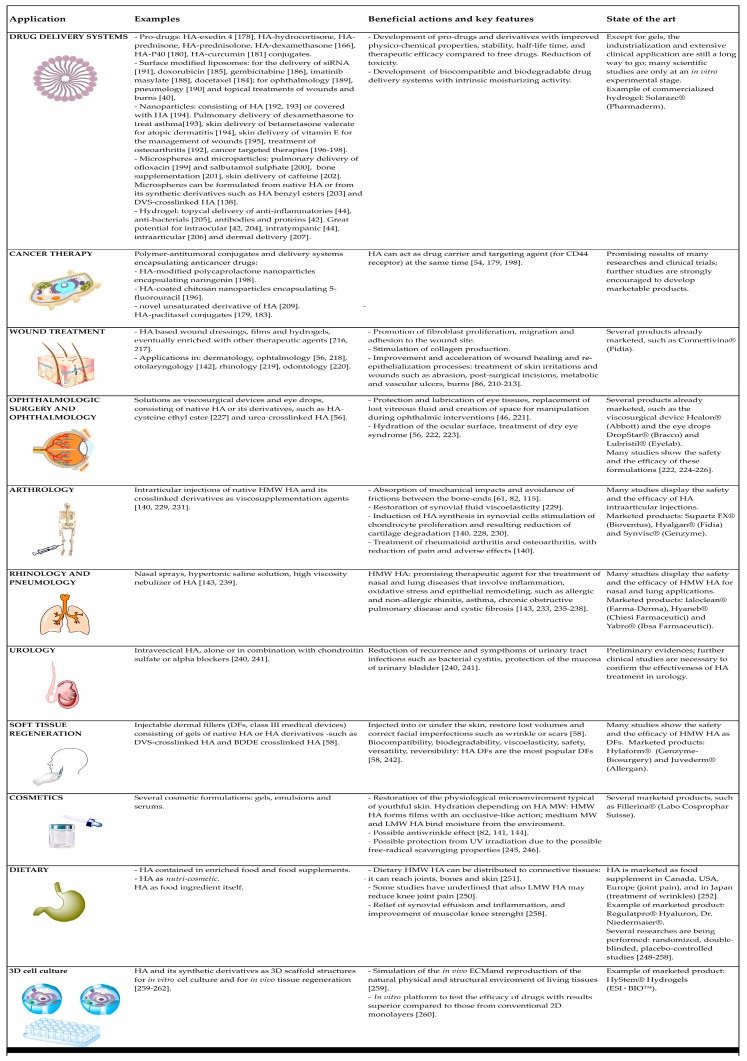
Summary of the medical, pharmaceutical, cosmetic and dietary applications of HA and its derivatives, reporting some examples, the beneficial actions, the key features and the state of the art.

Figure 7 summarize some of the most common medical, pharmaceutical, cosmetic and dietary applications of HA and its derivatives. The present review underlines the interest of academic and industrial research on HA: a comprehensive overview of this polymer is provided through the description of its structural, physico-chemical and hydrodynamic properties, occurrence, metabolism, biological roles, mechanisms of action, methods of production and derivatization, pharmaceutical, biomedical, food supplement and cosmetic applications.

During the last few decades, HA has shown great success due to its numerous and unique properties, such as biodegradability, biocompatibility, mucoadhesivity, hygroscopicity and viscoelasticity, and to the broad spectrum of chemical modifications that it can undergo, allowing the development of derivatives with specific targeting and long-lasting drug delivery. Several in vitro and in vivo studies have shown the beneficial actions of HA treatment, with anti-inflammatory, wound healing, chondroprotective, antiangiogenic, anti-ageing and immunosuppressive effects, among others [51,56,60,115]. This supported the development of a great number of HA-based commercial products: from native HA for ophthalmic and arthritic therapies, to food supplement, esthetic and cosmetic formulations. More recently, also some chemical derivatives of HA have received FDA approval and have been successfully introduced on the market, especially as DFs. As a proof of the great potential of this molecule in terms of real benefits for health, we also conducted (on 20 April 2018) a search on the patent database Questel (Paris, France), which resulted in the following table (Table 1) that illustrates the interest toward hyaluronan.

In our opinion, although hyaluronan displays a great number of potential applications, further investigations and technological improvements are required, as there are still some questions to be answered and some issues to be addressed. First of all, many aspects of HA metabolism, receptor clustering and affinity still need to be explored to understand the different biological actions that hyaluronan has through changes in MW fully. Additional insight needs to be gained in understanding whether there is a relation between HA size and localization and how concomitant use of different HA sizes may modulate signaling. The comprehension of all these mechanisms could provide opportunities to extend and improve hyaluronan pharmaceutical, biomedical, cosmetics and food supplements applications, obtaining more targeted effects. Towards this aim, the key mechanisms that control MW during HA biotechnological synthesis should be clarified to develop methods to produce more uniform size-defined HA. Additionally, progresses in metabolic engineering are necessary to improve HA yield and find biosynthetic strategies with good sustainability and acceptable production cost. Furthermore, the preparation of hyaluronan chemical derivatives needs to be optimized, using strategies such as one-pot reactions, chemo-selective synthesis, solvent-free methods and “click chemistry” approaches. Furthermore, the reproducibility of HA derivatives during scale-up, their pharmacokinetic and pharmacodynamic properties must be improved to allow their successful commercialization. Finally, all the HA-based next generation products, such as innovative crosslinked derivatives, polymer-drug conjugates and delivery systems, should be developed, enabling high biocompatibility, prolonged half-life and improved in situ permanence: hence, in vivo and clinical studies are required to characterize their safety and efficacy fully. Nevertheless, to date, recent in vitro research works have shown promising results, which open encouraging perspectives for safe and health uses of these novel derivatives: for example, HA-CL has displayed high biocompatibility towards human corneal and lung epithelial cell, as well as interesting anti-inflammatory, antioxidant and wound healing properties [56,263].

## Figures and Tables

**Table 1:** Data on hyaluronan patents resulting from the database Questel (Paris, France).

Total	13,684
Alive	8749
Dead	4935
**1st application year**	**unknown**
After 2015	2717
2011–2015	4568
2006–2010	2647
2001–2005	1694
Before 2001	2058

## Abbreviations

BDDE	butanediol-diglycidyl ether
CD44	cluster of differentiation-44
CDMT	2-chloro-dimethoxy-1,3,5-triazine
DFs	dermal fillers
DMF	dimethylformamide
DMTMM	4-(4,6-dimethoxy-1,3,5-triazin-2-yl)-4-methylmorpholinium
DMSO	dimethylsulfoxide
DVS	divinyl sulfone
ECM	extracellular matrix
EDC	*N*-(3-dimethylaminopropyl)-*N*′-ethylcarbodiimide hydrochloride
HA	hyaluronic acid; hyaluronate; hyaluronan
HA-CL	urea-crosslinked hyaluronic acid
HARE	hyaluronan receptor for endocytosis
HAS	hyaluronan synthases
H-bonds	hydrogen bonds
HYAL	hyaluronidases
HMW	high molecular weight
GAGs	glycosaminoglycans
GRAS	generally regarded as safe
LYVE1	lymphatic vessel endothelial hyaluronan receptor 1
LMW	low molecular weight
MW	molecular weight
NHS	*N*-hydroxysuccinimide
oHA	oligosaccharides of hyaluronic acid
RHAMM	receptor for HA-mediated cell motility
ROS	reactive oxygen species
TBA	tetrabutylammonium
TLRs	Toll-like receptors
